# Individual and Parental Factors of Adolescents’ mHealth App Use: Nationally Representative Cross-sectional Study

**DOI:** 10.2196/40340

**Published:** 2022-12-16

**Authors:** Hayriye Gulec, David Smahel

**Affiliations:** 1 Interdisciplinary Research Team on Internet and Society Faculty of Social Studies Masaryk University Brno Czech Republic

**Keywords:** mobile health, mHealth, eHealth literacy, parental mediation, health anxiety, sleep, body mass index, digital skills, phone attitudes, mobile phone

## Abstract

**Background:**

Knowledge of the characteristics of adolescents who use mobile health (mHealth) apps to monitor health and how these characteristics differ from those of app nonusers is limited.

**Objective:**

We aimed to determine mHealth app use based on adolescent and parental factors, including sociodemographics, digital skills, and health indicators, in a nationally representative sample of Czech adolescents (N=2500).

**Methods:**

Adolescents aged 11 to 16 years and one of their parents participated in an online survey in 2021. A professional research agency recruited the participants. Quotas were used to ensure the sample’s representativeness. The sociodemographic factors were the adolescents’ age, gender, and parental perceived financial security. The adolescents also provided information about their screen time, eHealth literacy, BMI, health anxiety, physical activity, and sleep quality. Parents reported their digital skills, mobile phone attitudes, and the mediation of their children’s online health information–seeking behaviors. We evaluated the differences between the users and nonusers of mHealth apps and identified the significant predictors of mHealth app use. Next, we separately examined how these factors were associated with the use of mHealth apps that track calorie intake or expenditure, number of steps, weight, or sports activity (eg, exercise, running, and working out), as well as other mHealth apps (eg, those that track sleep and heart rate).

**Results:**

More than half of the adolescents (1429/2455, 58.21%) reported using mHealth apps. App users were relatively older and, more often, girls. Apps that counted the number of steps were used most frequently, and adolescents whose parents reported higher perceived financial security used them more regularly. Overall, being older and physically active and having higher eHealth literacy skills were associated with using mHealth apps. Adolescents with higher BMI, health anxiety, and lower sleep quality more frequently used mHealth apps to track calorie intake or expenditure, weight, and health indicators. mHealth apps to track physical activity were used more regularly by girls. There was a positive association between parental mediation of online health information–seeking behaviors and adolescents’ mHealth app use.

**Conclusions:**

These findings demonstrated that older age, physical activity, and eHealth literacy skills were the common underlying factors of adolescents’ mHealth app use. We initially showed parents as significant role models for their children’s adoption of, and engagement with, mHealth apps when they actively mediate their online health information–seeking behaviors. Improving the eHealth literacy skills of adolescents through parental guidance might enhance health technology use in this population. Tracking eating behaviors, weight, and health were more prevalent for adolescents who reported higher BMI, health anxiety, and lower sleep quality. Future research studies should examine the determinants and health outcomes of adolescents’ mHealth app use longitudinally.

## Introduction

### Background

Adolescents use smartphones frequently, and the internet has become an integral part of their lives over the past decades [1,2]. Adolescents use their smartphones for, among other things, health-related purposes such as seeking online health information and using mobile apps to track their health [3,4]. Most studies on adolescents’ use of mobile health (mHealth) apps have evaluated the apps’ efficacy for disease management [5] and for improving health outcomes [6] such as physical activity [7,8], dietary behaviors [7], weight management [9], and sexual and reproductive health [10]. These mHealth apps were developed by researchers independently or in collaboration with app developers to guide the apps’ design and content. Otherwise, they were chosen from among the already available mHealth apps.

mHealth apps are mainly designed to enable users to pursue a healthy lifestyle, and they are primarily clustered around monitoring and managing features related to health, nutrition, and physical activity [11,12]. They allow users to track health-related features such as number of steps, heart rate, and sleep quality, and they support the monitoring and management of eating (eg, calorie intake or expenditure and weight management) and exercise (eg, fitness and sports activity) behaviors. Although studies on the efficacy of mHealth apps in improving health outcomes in adolescents are reported frequently, studies on representative samples that investigate the characteristics of adolescents who use mobile apps to monitor health and how these characteristics differ from those of app nonusers have scarcely been conducted. Determining the prevalence and correlates of adolescents’ use of mHealth apps can provide valuable information about their technology use to support a healthy lifestyle and wellness.

In a nationally representative sample of American adolescents, 70% of those aged between 14 and 22 years reported using mHealth apps in 2020 [4], and this percentage was slightly higher than that reported in a previous nationally representative sample of American adolescents (64%) in 2018 [3]. The most frequently used apps were related to fitness, sleep, menstruation, nutrition, and meditation [4]. Another study investigated the use of physical activity apps in a nationally representative sample of Finnish adolescents in 2017 [13]. The results showed that approximately half of the adolescents (52.8%) aged between 11 and 15 years owned these apps to track their physical activity; however, only 16.2% of these adolescents used the apps actively to track their physical activity. By contrast, almost half (47.4%) reported not owning these apps, and an additional 36.5% did not use the apps actively. A low proportion of mHealth app use (18.8%) was reported among Vietnamese youth aged 15 to 25 years in a cross-sectional study conducted in 2015 [14].

Studies that examined the sociodemographic correlates of mHealth app use among adolescents showed that older age and female sex were associated with using mobile apps for health [3,4,13]. Adolescents with a higher socioeconomic status were more likely to use physical activity trackers [13]. In addition, studies reported connections among mHealth app use, BMI, and physical activity. A higher BMI was related to the frequency of use of fitness and nutrition apps in a study with adolescents [15] and to the intention to use physical activity apps in another study with college students [16]. Adolescents who exercised at least once per week were more likely to use mHealth apps to track physical activity than those who never exercised or exercised rarely [13]. Although these findings provided preliminary evidence, only a few adolescent factors were investigated, limiting our understanding of mHealth app use in this population.

mHealth app use is mainly concerned with seeking health information and monitoring relevant health indicators to promote health and wellness [17]. Thus, the factors that promote online health information–seeking and health-monitoring behaviors might be potentially associated with the use of mobile apps for healthy lifestyle purposes. Therefore, in addition to sociodemographic characteristics, BMI, and physical activity, which were related to using mHealth apps in previous studies [4,13,15], we examined the roles of adolescents’ eHealth literacy, screen time, health anxiety, and sleep quality in this study. eHealth literacy refers to the knowledge and skills related to obtaining, understanding, and evaluating online health information and pursuing it to promote health and prevent illnesses [18,19]. Previous research identified an association between higher eHealth literacy and health-promoting behaviors in adolescents [20] and between higher health literacy and using mHealth apps in adults [21]. Therefore, we examined the role of eHealth literacy in the mHealth app use of adolescents. We also examined the role of adolescents’ screen time because the time spent online is associated with, in general, searching for online information [22]. In addition, we examined the roles of health anxiety and sleep quality because their physical manifestations can be tracked by mobile apps that monitor health indicators such as heart rate and sleep.

Thus far, most studies have focused on adolescent-related factors to determine the use of mHealth apps and neglected to examine the social determinants of such behaviors; for instance, there is currently a lack of evidence for the role of parents in their adolescent children’s adoption of mHealth apps. Nevertheless, parents model their children’s online behaviors [23,24], and parental factors connected with adolescents’ adoption of new technologies deserve further research. Parental mediation refers to the behaviors and strategies applied by parents to regulate their children’s media use [25]. Previous studies showed that parental mediation was associated with adolescents’ online health behaviors and eHealth literacy skills [26,27]. In this study, we focused on the parental mediation of online health information–seeking behaviors, which refers to parents’ involvement in enhancing adolescents’ eHealth literacy skills for assessing the quality and trustworthiness of online health information. eHealth literacy is closely connected to health-promoting behaviors [20]. Therefore, we expect adolescents who receive parental mediation to improve their eHealth literacy skills to be more likely to adopt digital technologies that promote health, including mHealth apps. We also examined the role of parental digital skills in adolescents’ adoption of mHealth apps. Recent studies highlight the significance of parental factors in adolescents’ technology use [28]. In addition, we know from previous research that parental digital skills are associated with increased opportunities for adolescents [25]. Therefore, we expect adolescents with higher parental digital skills to be more likely to explore health-promoting technologies such as mHealth apps. Finally, we examined parents’ attitudes toward their children’s use of mobile phones because parental mobile phone attitudes might influence the amount of time that adolescents spend online and the variety of online activities they engage in with their mobile phones [29].

There are various mHealth apps available in the market to monitor and manage a healthy lifestyle (eg, health-related features, nutrition, and physical activity). Nevertheless, the frequency with which adolescents use these different mHealth apps and whether the use patterns differ depending on adolescent and parental factors are relatively unknown. Previous research demonstrated an association between higher BMI and fitness and nutrition apps [15,16] and between higher levels of physical activity and fitness-related apps [13]. Nevertheless, further research is required to understand app use in this population; for instance, factors such as health anxiety and lower-quality sleep may be more closely related to using mHealth apps to track health-related features. By contrast, factors such as adolescents’ eHealth literacy, screen time, and parental mediation of their online health information–seeking behaviors might not differentiate among the user groups. Thus, they might explain adolescents’ general adoption and use patterns of new technologies for health management purposes. Therefore, we evaluated the roles of adolescent and parental factors separately in adolescents’ use of different types of mHealth apps in this study.

### Objectives

This study examined the prevalence of mHealth app use in a nationally representative sample of Czech adolescents. The mobile internet penetration rate in the Czech Republic (91%) is similar to the European Union average (92%) [30], and 82% of the Czech children aged 9 to 16 years use their smartphones to access the internet at least daily [2]. This study provides the initial evidence from a nationally representative sample concerning adolescents’ mHealth app use in Europe.

We evaluated the differences between users and nonusers of mHealth apps and identified the significant predictors of mHealth app use based on sociodemographic and selected adolescent and parental factors. Next, we separately examined how these factors were associated with use of mHealth apps that track (1) calorie intake or expenditure, (2) number of steps, (3) weight, or (4) sports activity (eg, exercise, running, and working out), as well as (5) other mHealth apps (eg, those that track sleep and heart rate). No previous study has explored the role of parents in the mHealth app use of adolescents. In addition, to the best of our knowledge, this study is the first to examine sociodemographic, adolescent, and parental factors together in the mHealth app use of adolescents and how these factors might be associated with the use of different types of apps.

## Methods

### Recruitment

This study recruited a nationally representative sample of 2500 Czech adolescents (1250/2500, 50% girls) aged 11 to 16 (mean 13.43, SD 1.70) years and 2492 caregivers, of whom 1589 (63.76%) were women aged 18 to 74 (mean 42.75, SD 7) years. The data constitute the first wave of a longitudinal study that examined the impact of information and communication technologies on the well-being of adolescents. A professional agency recruited the participants and conducted the online data collection in June 2021 as part of the Future project (Modeling the Future: Understanding the Impact of Technology on Adolescents’ Well-being). The target group for eligibility was Czech households, with 1 parent or caregiver and 1 adolescent (aged 11-16 years) who would fill out the questionnaire online. The agency selected eligible participants for the final sample from a combined pool of 3 Czech online panels (approximately 165,000 panelists) and 980 newly recruited households. Quota sampling was used with equal distributions for the adolescents’ gender and age. The sampling procedure considered household income, administrative region according to the Nomenclature of Territorial Units for Statistics, and municipality size to ensure a proportional representation of Czech households with children. Before the data collection, cognitive testing in the form of semistructured interviews was conducted to test the comprehension of the questionnaires by respondents from different age groups. In addition, pilot testing was conducted on 195 adolescents and one of their parents to check the data distributions in all variables and to determine the dimensions and internal reliabilities of the scales. Adolescents and parents filled out an online questionnaire at their homes. Only 1 adolescent and 1 parent were recruited from each household. The computer-assisted web interviewing method was used. The agency obtained written informed consent from the adolescents and their parents before participation. Before they filled in the questionnaires, the participants were briefed about the survey’s aim, anonymity, and the possibility of refusing to participate. They were also informed about the possibility of answering any question with *I don’t know* or *I prefer not to say* options. The agency checked the completion times for the questionnaires and monitored the consistency of the entries between the parent and the child. The agency also applied quality checks on the collected data and removed respondents with poor data quality from the final data set.

### Ethics Approval

The research ethics committee of Masaryk University approved this study (EKV-2018-068).

### Measures

#### Sociodemographic Characteristics

Both adolescents and parents reported their gender, and they responded to an open-response question to indicate their age. The parents provided information about their perceived financial security. The question was as follows: *How does your household manage its total monthly income?* The response scale included (1) *with great difficulty*, (2) *with difficulty*, (3) *with minor difficulty*, (4) *somewhat easily*, (5) *easily*, and (6) *very easily*. Parental perceived financial security was used as an indicator of familial affluence.

#### Screen Time

Adolescents reported the time they spent using computers (laptop or desktop) and mobile phones or tablet devices, as well as watching television, including DVDs and Netflix, during usual weekdays. The screen time, calculated in hours, was obtained by adding the time spent using each device.

#### eHealth Literacy

eHealth literacy was measured using the eHealth Literacy Scale [31]. Adolescents reported on their knowledge of online health information sources (ie, *I know what health resources are available on the internet*), how to navigate the internet to obtain answers to health-related questions (ie, *I know where to find helpful health resources on the internet*), and their perceived skills to evaluate the quality of online health information (ie, *I can tell high-quality health resources from low-quality health resources on the internet*). The response scale ranged from 1 (strongly disagree) to 5 (strongly agree). Higher scores indicated better eHealth literacy skills. The internal consistency was adequate (Cronbach α=.89).

#### BMI Calculation

The BMI was calculated from the adolescents’ self-reported answers to open-ended questions about their height (in centimeters) and weight (in kilograms).

#### Health Anxiety

Health anxiety was measured with a modified version of the Multidimensional Inventory of Hypochondriacal Traits [32], which measures self-reported health anxiety in cognitive, behavioral, perceptual, and affective domains. In this study, 4 items from the affective domain to assess hypochondriacal worry were used. Adolescents reported how much the following statements applied to them: *I worry a lot about my health*; *When I experience pain, I fear I may be ill*; *Reading articles about disease makes me worry about my health*; and *I am concerned with the possibility of being diagnosed with a serious disease*. The response options included (1) *very untrue*, (2) *somewhat untrue*, (3) *neutral*, (4) *somewhat true*, and (5) *very true*. Higher scores indicated increased health anxiety. The internal consistency was adequate (Cronbach α=.85).

#### Physical Activity

Adolescents responded to the following question: *How many of your free-time hours each week do you usually exercise to the extent that you sweat and feel shortness of breath (excluding the compulsory physical education at school)?* The response options included (1) *less than half an hour per week*, (2) *about half an hour per week*, (3) *about 1 hour per week*, (4) *about 2-3 hours per week*, (5) *about 4-6 hours per week*, and (6) *about 7 hours or more per week*.

#### Sleep Quality

Sleep quality was addressed with the following question: *In the last month, how would you rate your overall sleep quality?* The response options were (1) *very bad*, (2) *fairly bad*, (3) *fairly good*, and (4) *very good*. Lower scores were indicative of a worse quality of sleep.

#### Parental Mediation of Online Health Information–Seeking Behaviors

Parental mediation of adolescents’ online health information–seeking behaviors was measured with 4 items adapted from the eHealth Literacy Scale [31]. The parents reported the frequency of their involvement in discussing the trustworthiness and quality of online health information with their adolescent children in the preceding few months. The items consisted of how often parents discussed with their children the following topics: *Whether we can trust health-related information on the internet*, *How we can tell that health-related information on the internet is true or false*, *How we can tell that the author of health-related information on the internet is trustworthy*, and *How we can evaluate the quality of health-related information on the internet*. The response options included (1) *never*, (2) *a few times at most*, (3) *several times a month*, (4) *several times a week*, (5) *every day*, and (6) *several times a day*. The internal consistency was adequate (Cronbach α=.94).

#### Parental Digital Skills

Parents evaluated how advanced they were in terms of using (1) computers, (2) the internet, and (3) smartphones. The response options ranged from 1=*beginner* to 8=*expert*. Parental digital skills were determined by the average score of the items.

#### Parental Mobile Phone Attitudes

Parental mobile phone attitudes were measured by adapting the 5 items from a previous study [33] that evaluated parental attitudes toward their children’s internet use. We changed the word “internet” to “mobile phones” in this study. The items consisted of the following statements: *Mobile phones should be used by the whole family*, *Mobile phones harm children in learning*, *Mobile phones will enhance the overall development of a child*, *Mobile phones harm children in developing thinking skills*, and *Children need to learn to use mobile phones now to be successful in the future*. The parents indicated their attitudes toward their children’s mobile phone use on a scale that ranged from 1=*strongly disagree* to 5=*strongly agree*. The reverse items were recoded so that higher numbers represented more positive attitudes. The internal consistency was adequate (Cronbach α=.67).

#### Use of mHealth App by Adolescents

Adolescents’ app use was determined by their response to the following question: *You can use various applications on your phone, tablet, and other devices. Do you use applications to monitor health and exercise (e.g., counting steps, tracking calories, weight, sports activities, eating/drinking, stress, sleep)?* The response options were (1) *No* and (2) *Yes*. Those adolescents who indicated that they were using mHealth apps responded to an additional question about the frequency of using different apps: *Such applications can be used to monitor or record data in various areas of health. How often have you used them in the last six months in the following areas?* The use frequency was assessed for (1) calorie intake or expenditure, (2) number of steps, (3) weight, (4) sports activity (eg, exercise, running, and working out), and (5) other health apps (eg, those that track sleep and heart rate). The response options included (1) *never*, (2) *once*, (3) *no more than a few times*, (4) *several times a month*, (5) *several times a week*, (6), *daily*, and (7) *several times a day*.

### Statistical Analysis

Descriptive statistics were run for the sociodemographic, adolescent, and parental factors, including means, SDs, and frequencies for the whole sample, app users, and nonusers. The differences in the studied variables between app users and nonusers were analyzed using independent sample *t* tests and chi-square difference tests. A 2-tailed α=.05 was applied to statistical testing. The effect sizes were calculated using the Hedges *g* correction for independent sample *t* tests and the φ coefficient for chi-square difference tests. Hierarchical logistic regression analysis examined the significant predictors of mHealth app use. We entered the sociodemographic factors in the first step, followed by entering adolescent factors in the second step and parental factors in the third step. We presented the adolescents’ use frequency of different apps and conducted separate hierarchical regression analyses to identify the significant factors related to the adolescents’ use of each app type. The analyses were run using SPSS software (version 28.0; IBM Corp) [34]. Mahalanobis distances were computed to check multiple outliers, and the data obtained from 1.24% (31/2500) of the participants with significant Mahalanobis distance values at *P*<.001 were deleted.

## Results

### Prevalence of mHealth App Use and Sample Characteristics

The total sample size consisted of 2469 adolescents and one of their parents. More than half of the adolescents (1429/2455, 58.21%) reported using mHealth apps on their devices. We examined the sociodemographic, adolescent, and parental factors of the whole sample and the differences between app users and nonusers (Table 1). Girls accounted for 49.9% (1232/2469) of the sample, and the mean age of the participants was 13.42 (SD 1.70) years. The caregivers (n=2333) were aged between 18 and 74 (mean 42.74, SD 7.01) years, and most of the caregivers who responded to the questionnaires were women (1567/2461, 63.67%). More than half of the households (1369/2449, 55.9%) reported managing their monthly income somewhat easily or easily. At the same time, 24.66% (604/2449) reported managing their household income with minor difficulty, 6.45% (158/2449) with difficulty, and 2.9% (71/2449) with great difficulty, whereas 10.09% (247/2449) of the participants reported managing their household income very easily.

**Table 1:** Sociodemographic, adolescent, and parental factors of the sample.

Variables		Total sample (N=2469)			App users^a^ (n=1429)			App nonusers (n=1026)			*P* value			Effect size		
**Sociodemographic factors**																
	Age (years), mean (SD)	13.42 (1.70)			13.61 (1.65)			13.16 (1.72)			<.001^b^			0.267		
	Gender, girl, n (%)	1232 (49.9)			759 (53.11)			466 (45.42)			<.001^b^			0.076		
	Parental perceived financial security, mean (SD)	3.95 (1.17)			4 (1.16)			3.87 (1.18)			.01^b^			0.106		
**Adolescent factors, mean (SD)**																
	Screen time in hours			7.25 (4.05)			7.4 (4)			7.03 (4.1)			.03^b^			0.092
	eHealth literacy			3.34 (0.87)			3.46 (0.83)			3.17 (0.9)			<.001^b^			0.329
	BMI			20.43 (3.84)			20.48 (3.56)			20.38 (4.19)			.55			0.026
	Health anxiety			2.47 (0.97)			2.54 (0.98)			2.35 (0.94)			<.001^b^			0.195
	Physical activity			4.35 (2.15)			4.66 (2.11)			3.91 (2.14)			<.001^b^			0.356
	Sleep quality			3.24 (0.64)			3.21 (0.66)			3.28 (0.62)			.01^b^			0.106
**Parental factors, mean (SD)**																
	Parental OHIS^c^ mediation		2.34 (1.03)			2.46 (1.06)			2.16 (0.96)			<.001^b^			0.289	
	Parental digital skills		5.59 (1.35)			5.63 (1.34)			5.53 (1.37)			.08			0.073	
	Parental mobile phone attitudes		3.26 (0.67)			3.28 (0.65)			3.24 (0.7)			.08			0.072	

^a^App use was determined by “Yes” or “No” responses to the item assessing the use of mobile health apps by adolescents. The number of app users and nonusers is less than the total sample size because of missing values on this variable.

^b^Significant *P* value.

^c^OHIS: online health information seeking.

Adolescents’ screen time on weekdays was, on average, 7 hours and 15 minutes (SD 4 hours 3 minutes). The mean BMI was in the normal range. The adolescents evaluated their eHealth literacy skills rather positively, had a medium level of health anxiety, and their sleep quality ranged between *fairly good* and *very good*. They were physically active between *2 to 3 hours per week* and *4 to 6 hours per week*. The mean parental mediation of online health information–seeking behaviors was between *several times a month* and *several times a week*. Parents evaluated their digital skills highly and had somewhat positive attitudes toward their adolescent children’s use of mobile phones.

### Comparison Between App Users and Nonusers

There were statistically significant differences between app users and nonusers regarding age, gender, and parental perceived financial security (Table 1). App users were relatively older (t_2453_=–6.47; *P*<.001) and, more often, girls (*χ*^2^_1_=14.1; *P*<.001), and their parents reported higher perceived financial security (t_2433_=–2.58; *P*<.001). All adolescent factors were significantly different between app users and nonusers, except for BMI (t_2189_=–0.59; *P*<.55). App users reported significantly longer screen time (t_2440_=–2.24; *P*=.02) and better eHealth literacy skills (t_2445_=–8.04; *P*<.001). They were more likely to report higher health anxiety (t_2453_=–4.76; *P*<.001) and lower sleep quality (t_2444_=2.58; *P*<.001). In addition, they were physically more active than app nonusers (t_2407_=–8.6; *P*<.001). As for the parental factors, only the parental online health information–seeking mediation differed between the groups. The parents of app users reported a higher frequency of mediating their adolescent children’s online health information–seeking behaviors than the parents of app nonusers (t_2422_=–7.02; *P*<.001). Although significant differences were observed between app users and nonusers, the effect sizes for the observed differences were small for age, eHealth literacy, and parental online health information–seeking mediation, and they were negligible for gender, parental perceived financial security, screen time, health anxiety, and sleep quality.

The hierarchical logistic regression analysis examined the correlates of mHealth app use by the adolescents (Table 2). The results demonstrated that female sex and older age were significantly associated with mHealth app use, but parental perceived financial security was not. After controlling for sociodemographic factors, the adolescent factors that explained mHealth app use were physical activity, eHealth literacy, health anxiety, and sleep quality. Adolescents who were physically more active and had higher eHealth literacy skills were more likely to use mHealth apps. Using mHealth apps was associated with higher health anxiety and lower sleep quality. There were no significant relationships between mHealth app use and the adolescents’ BMI and screen time. In the final step, the roles of parental factors were examined, controlling for sociodemographic and adolescent factors. The results demonstrated that adolescents whose parents were more likely to mediate their children’s online health information–seeking behaviors were more likely to use mHealth apps. Factors related to parental digital skills and mobile phone attitudes were not significantly associated with mHealth app use of the adolescents.

#### Factors of mHealth App Use by Type of App

The remaining analyses focused on the subsample of adolescents who reported using mHealth apps (n=1429). First, we examined the frequency of mHealth app use for each mHealth app type (Table 3). The apps that counted the number of steps were the most frequently used mHealth apps for adolescents: 48.7% (693/1423) reported using them daily or several times a day. These were followed by mHealth apps that tracked health indicators such as heart rate and sleep quality: 21.67% (308/1421) used them daily or several times a day. Sports-related mHealth apps that track exercise, fitness, and physical activity were used daily or several times a day by 17.36% (247/1423) of the adolescents. The least frequently used mHealth apps were those that tracked calorie intake or expenditure and weight. mHealth apps related to calorie intake or expenditure were used by 12.22% (173/1416) of the adolescents daily or several times a day, whereas 6.86% (97/1415) of the adolescents used mHealth apps to track weight daily or several times a day.

**Table 2:** Sociodemographic, adolescent, and parental factors related to adolescents’ mobile health app use.

Variable					B		SE		*P* value		Exp (B) (95% CI)
**Block 1 (sociodemographic factors)^a^**											
		Age (years)		0.13		0.03		<.001^b^		1.14 (1.08-1.21)	
		Gender		–0.35		0.09		<.001^b^		0.71 (0.59-0.85)	
		Parental perceived financial security		0.08		0.04		.07		1.08 (0.99-1.17)	
**Block 2 (adolescent factors)^c^**											
			Screen time in hours	–0.00		0.01		.76		1.0 (0.97-1.02)	
			eHealth literacy	0.23		0.06		<.001^b^		1.26 (1.13-1.41)	
			BMI	0.01		0.01		.52		1.01 (0.98-1.03)	
			Health anxiety	0.15		0.05		.003^b^		1.16 (1.05-1.28)	
			Physical activity	0.17		0.02		<.001^b^		1.18 (1.13-1.24)	
			Sleep quality	–0.16		0.08		.03^b^		0.85 (0.73-0.99)	
**Block 3 (parental factors)^d^**											
	Parental OHIS^e^ mediation			0.19		0.05		<.001^b^		1.21 (1.10-1.34)	
	Parental digital skills			0.02		0.04		.57		1.02 (0.95-1.09)	
	Parental mobile phone attitudes			0.02		0.07		.80		1.02 (0.89-1.17)	

^a^Nagelkerke *R*^2^=0.03.

^b^Significant *P* value.

^c^Nagelkerke *R*^2^=0.10.

^d^Nagelkerke *R*^2^=0.11.

^e^OHIS: online health information seeking.

**Table 3:** Adolescents’ use of different types of mobile health (mHealth) apps (N=1429).

Type of mHealth app	Never, n (%)	Once, n (%)	A few times at most, n (%)	Several times a month, n (%)	Several times a week, n (%)	Daily, n (%)	Several times a day, n (%)
Calorie intake or expenditure (n=1416)	465 (32.84)	131 (9.25)	324 (22.88)	167 (11.79)	156 (11.02)	136 (9.61)	37 (2.61)
Number of steps (n=1423)	61 (4.29)	40 (2.81)	143 (10.05)	232 (16.3)	254 (17.85)	515 (36.19)	178 (12.51)
Weight (n=1415)	498 (35.19)	139 (9.82)	304 (21.48)	231 (16.33)	146 (10.32)	74 (5.23)	23 (1.63)
Sports activity (n=1423)	212 (14.9)	87 (6.11)	273 (19.19)	298 (20.94)	306 (21.5)	178 (12.51)	69 (4.85)
Health (n=1421)	329 (23.15)	98 (6.9)	243 (17.1)	211 (14.85)	232 (16.33)	249 (17.52)	59 (4.15)

To identify the sociodemographic, adolescent, and parental factors related to mHealth app use, we conducted separate hierarchical linear regression analyses by each app type (Table 4). The results demonstrated that, regardless of the kind of app, older age was associated with a higher frequency of mHealth app use. Gender was significantly associated with the use of mHealth apps for physical activity (ie, number of steps and sports activity). Girls used these mHealth apps more frequently. Higher perceived financial security by parents was associated with the frequency of using mHealth apps that tracked the number of steps. After controlling for sociodemographic characteristics, the adolescent factors associated with using all mHealth app types were the adolescents’ eHealth literacy skills and their level of physical activity. mHealth apps were used more frequently by adolescents who had higher eHealth literacy skills and who were physically more active. Higher BMI was associated with the use of mHealth apps to manage calorie intake or expenditure and weight. Adolescents with lower sleep quality and higher health anxiety used mHealth apps to track weight and health indicators more frequently (eg, heart rate and sleep quality). The adolescents’ screen time was not significantly associated with the use of mHealth apps. After controlling for sociodemographic and adolescent factors, the only parental factor related to the adolescents’ mHealth app use was parental online health information–seeking mediation. Adolescents whose parents reported a higher frequency of mediating their children’s online health information–seeking behaviors used mHealth apps to track calorie intake or expenditure, weight, physical activity, and health more frequently. Parental digital skills and mobile phone attitudes were not significantly associated with the use of mHealth apps.

**Table 4:** Sociodemographic, adolescent, and parental factors related to adolescents’ frequency of mobile health app use by type of app (N=1429).

		Calorie intake or expenditure (n=1416)					Number of steps (n=1423)							Weight (n=1415)							Sports activity (n=1423)							Health (n=1421)					
		β		*P* value			β			*P* value			β				*P* value			β				*P* value			β				*P* value		
**Block 1 (sociodemographic factors)^a^**																																	
	Age (years)		.09			.003^b^			.07			.03^b^				.07			.02^b^				.12			<.001^b^				.08			.005^b^
	Gender		–.05			.08			–.07			.02^b^				–.05			.08				–.06			.03^b^				–.00			.94
	Parental perceived financial security		.00			.99			.07			.01^b^				–.05			.08				–.01			.62				.02			.50
**Block 2 (adolescent factors)^c^**																																	
	Screen time	.03			.27			–.03			.40				.04			.19				–.03			.28				–.02			.41	
	eHealth literacy	.10			<.001^b^			.06			.04^b^				.09			.003^b^				.11			<.001^b^				.08			.008^b^	
	BMI	.10			<.001^b^			.03			.32				.09			.001^b^				.05			.01				–.00			.87	
	Health anxiety	.05			.07			–.00			.89				.11			<.001^b^				–.01			.65				.08			.004^b^	
	Physical activity	.14			<.001^b^			.19			<.001^b^				.12			<.001^b^				.31			<.001^b^				.20			<.001^b^	
	Sleep quality	–.04			.17			–.04			.18				–.06			.02^b^				–.04			.12				–.06			.03^b^	
**Block 3 (parental factors)^d^**																																	
	Parental OHIS^e^ mediation	.17			<.001^b^			.00			.99				.24			<.001^b^				.14			<.001^b^				.16			<.001^b^	
	Parental digital skills	–.02			.50			.05			.07				.02			.56				.02			.59				.02			.51	
	Parental mobile phone attitudes	.02			.59			–.03			.27				.01			.73				–.00			.95				.02			.56	

^a^Calorie intake or expenditure: *R*^2^=0.02, number of steps: *R*^2^=0.02, weight: *R*^2^=0.02, sports activity: *R*^2^=0.02, and health: *R*^2^=0.01.

^b^Significant *P* value.

^c^Calorie intake or expenditure: *R*^2^=0.08, number of steps: *R*^2^=0.06, weight: *R*^2^=0.09, sports activity: *R*^2^=0.13, and health: *R*^2^=0.08.

^d^Calorie intake or expenditure: *R*^2^=0.10, number of steps: *R*^2^=0.06, weight: *R*^2^=0.15, sports activity: *R*^2^=0.15, and health: *R*^2^=0.11.

^e^OHIS: online health information seeking.

## Discussion

### Principal Findings

#### Overview

This study focused on mHealth app use by adolescents. It examined the sociodemographic, adolescent, and parental factors for mHealth app use and how they were related to the frequency of using different types of mHealth apps in a nationally representative sample of Czech adolescents. Previous research identified an association between mHealth app use and adolescents’ age, gender, socioeconomic status, BMI, and physical activity [3,4,13,16]. In addition to these variables, we investigated whether adolescents’ eHealth literacy, screen time, health anxiety, and sleep quality were associated with their mHealth app use. Furthermore, we initially examined parental factors, including the parents’ digital skills and mobile phone attitudes as well as their mediation of their children’s online health information–seeking behaviors. To the best of our knowledge, this is the first study to explore the role of parents in their adolescent children’s use of mHealth apps. It provides a more complex and comprehensive understanding of mHealth app use by adolescents.

#### Prevalence of mHealth App Use

More than half of the adolescents (1429/2455, 58.21%) reported using mHealth apps on their devices. The mHealth app use rate was higher than that in a previous study that examined the ownership of physical activity apps (52.8%) in a nationally representative sample of Finnish adolescents in 2017 [13], but it was lower than the use rates in nationally representative samples of American adolescents, who reported use rates of 69% in 2020 and 64% in 2018 [3,4]. Similar to the studies conducted on the American samples, we asked adolescents to report whether they used any mHealth apps on their devices. The difference in the reported rates, albeit lower (by approximately 10%), might be related to the sample characteristics; for instance, the American samples included participants aged between 14 and 22 years, whereas the age range in the Czech sample was between 11 and 16 years. Having emerging adults who are frequent users of mHealth apps [35] as participants might have contributed to the increased use rates in the American samples. Nevertheless, future research might consider the country-level differences and how they might be associated with adopting new technologies for health promotion purposes among adolescents.

#### Comparison Between App Users and Nonusers

The findings demonstrated that adolescents’ age and gender differed between the groups of app users and nonusers. App users were more often adolescent girls and older. These findings were in line with previous research that indicated sociodemographic differences for age and gender between mHealth app users and nonusers in representative adolescent samples [3,4,13]. The increased use of health technologies in older adolescents and girls could be associated with the higher frequency of online health information–seeking behaviors reported for this segment of adolescents [27]. To interpret our findings further, it should be noted that the between-group comparisons revealed a small effect size for age, whereas the effect size for gender was negligible (Table 1). Nevertheless, both variables significantly predicted adolescents’ mHealth app use when controlling for adolescent and parental factors in the logistic regression analysis (Table 2). Therefore, their roles should be considered in understanding the mHealth app use of adolescents.

A previous study that used an objective measure to determine socioeconomic status reported higher family affluence for adolescents who used physical activity apps than for those who did not use such apps [13]. We assessed the perceived financial security of parents as an indicator of familial affluence in this study. The results revealed that the parents of app users perceived higher financial security than the parents of nonusers (Table 1). However, the effect size for the observed difference was too small to be considered significant. Furthermore, it did not significantly predict mHealth app use in the logistic regression analysis when adolescent and parental variables were also included in the model (Table 2). Thus, contrary to our expectation, family affluence did not play a significant role in explaining the mHealth app uptake of the adolescents. Further research can examine whether similar findings would be observed when objective criteria are used to determine socioeconomic status.

In this study, mHealth app users were physically more active than nonusers. A previous study demonstrated higher use of physical activity apps with increasing physical activity among adolescents [13]. The adolescents in our study were asked to report whether they used any mHealth apps on their devices. Thus, our findings indicate that higher levels of physical activity (such as taking part in sports activities) are related to adolescents’ adoption of mHealth apps. Health consciousness could be a possible explanation for the connection between physical activity and mHealth app use. Health-conscious individuals are more aware of their health conditions, are more motivated to stay healthy, and perceive higher personal responsibility for their health [36]. Higher health consciousness is related to healthier lifestyle behaviors such as regular physical activity [37,38].

We found initial evidence for the significant role of higher eHealth literacy skills in differentiating between mHealth app users and nonusers. Previous studies in representative adult samples showed that those with higher health literacy skills were more likely to use mHealth apps [17,21]. This study found a similar pattern and showed that adolescents who used mHealth apps were more skillful in understanding and using online health information for health purposes than adolescents who did not use mHealth apps. These findings suggest that the digital disparities in health literacy skills, which limited the use of technology for health purposes in adult samples, were similarly related to the limited use of mHealth apps among adolescents. Therefore, enhancing eHealth literacy skills could be a significant route to the reduction of digital disparities concerning mHealth app use.

This study newly showed that mHealth app users were more likely to score worse on health anxiety and sleep quality than nonusers. It seems that some adolescents could use mobile apps to improve their health status. However, it should be noted that the effect sizes were negligible when comparing app users with nonusers (Table 1), and the odds ratios were closer to 1 when predicting the probability of mHealth app use in the logistic regression analysis (Table 2). Therefore, our findings should be replicated before they can be generalized. Further research can examine the specific types of apps used by these users and how app use is related to their health outcomes; for instance, using an app to diagnose bodily symptoms was associated with increased health anxiety in a previous study [39]. There were no significant differences in BMI between the groups. A previous study with adolescents reported a higher use frequency for nutrition and physical activity apps with increasing BMI [15]. The BMI of app users and nonusers was in the normal range in this study. Future research could investigate whether app users and nonusers would differ in underweight, normal weight, overweight, and obese BMI categories.

App users reported longer screen time than nonusers (Table 1), meaning that app users used digital devices more than nonusers. Nevertheless, the effect size was negligible, and mere duration of digital activity did not significantly predict mHealth app use after controlling for sociodemographic factors (Table 2). Future studies could determine whether certain online activities are related to mHealth app use.

This study is the first to consider parents’ behaviors, skills, and attitudes in adolescents’ mHealth app use. The only parental variable that differed between the groups was the parental mediation of online health information–seeking behaviors. The parents of app users were more actively involved in mediating their children’s online health information–seeking behaviors than the parents of nonusers. In other words, adolescents whose parents discussed the reliability and trustworthiness of online health information more frequently with their children were more likely to use mHealth apps. This finding supports previous research that demonstrated the significant role of parental mediation of internet use in adolescents’ online health behaviors and eHealth literacy skills [26,27]. Parental involvement in enhancing the eHealth literacy skills of adolescents could be a significant social determinant for their health-promoting behaviors in the digital space. Therefore, improving the eHealth literacy skills of parents and encouraging their involvement in guiding their children’s critical appraisal skills to evaluate online health information could promote adolescents’ use of online health information and technologies.

#### Factors of mHealth App Use by Type of App

This study contributes to the literature by revealing the factors of adolescents’ app use by type of app. We separately examined the roles of sociodemographic, adolescent, and parental factors in the frequency of using mHealth apps for tracking (1) calorie intake or expenditure, (2) number of steps, (3) weight, (4) sports activity (eg, exercise, running, and working out), and (5) other health apps (eg, those that track sleep and heart rate).

The adolescents most frequently reported using mHealth apps to count the number of steps, followed by apps that track health and physical activity. mHealth apps that track the number of steps are often built-in apps delivered in major mobile phone companies’ latest smartphones. Therefore, one of the possible explanations for the frequent use of these mHealth apps might be related to their ready availability. Previous studies of American adolescents reported fitness apps to be the most commonly used [3,4]. However, in their analyses, they did not examine the apps that tracked the number of steps separately, and they did not report on the factors related to their use.

Higher levels of physical activity and older age were associated with adolescents’ use of physical activity apps in a previous study [13]. Our findings expanded on this and showed that higher levels of physical activity and older age were associated with using apps to track calorie intake or expenditure, weight, exercise, health, and the number of steps. Furthermore, we initially showed that adolescents with higher eHealth literacy skills used mHealth apps more frequently. Persistence is essential for achieving the long-term benefits of using mHealth apps [40]. Therefore, these findings are novel because they indicated that persistence in using different types of apps could be associated with the same underlying factors. Future research could investigate whether the general factors identified in this study explain the use of other types of mHealth apps such as those related to meditation or menstruation.

mHealth apps that track calorie intake or expenditure and weight were more frequently used by adolescents who had a higher BMI. A previous study demonstrated an association between higher BMI and adolescents’ use of fitness and nutrition apps [15]. Our findings supported the link between BMI and tracking eating behaviors and weight. However, we could not show a connection between adolescents’ BMI and fitness app use. Instead, using fitness-related mHealth apps was significantly associated with the female sex. Adolescent girls tracked the number of steps and sports activities more regularly than boys. Body dissatisfaction is more prevalent in adolescent girls than boys [41]. Thus, the extent to which the use of physical activity apps by adolescent girls is connected to an effort to control their bodies or improve their health needs to be investigated.

Adolescents with higher health anxiety and lower sleep quality tracked their weight, heart rate, and sleep quality more frequently with mHealth apps. Studies indicate an association among excess BMI, health anxiety, and somatic complaints [42]. Similarly, the sleep-wake cycle and heart rate variability are related [43]. There is also an association between excess BMI and the quality and duration of sleep [44]. Therefore, it is reasonable to find an association among adolescents’ health anxiety, poor sleep quality, and mHealth app use to track BMI, heart rate, and sleep quality. We also found that adolescents with higher BMI used mHealth apps to track eating behaviors and weight more frequently. Altogether, these findings suggest an association between poorer well-being as related to BMI, health anxiety, and sleep quality and the monitoring of indicators such as eating behaviors, weight, heart rate, and sleep quality with mHealth apps. Whether the frequent use of mHealth apps is related to better well-being or worsened outcomes because of the increasing risk for certain conditions (eg, eating disorders and somatic complaints) in these adolescents should be investigated.

After controlling for sociodemographic and adolescent factors, the only parental variable associated with app use was the parental mediation of online health information–seeking behaviors. Adolescents who received parental mediation of their online health information–seeking behaviors more frequently used mHealth apps to track calorie intake or expenditure, weight, exercise, and health. However, parental mediation did not significantly predict the frequency of using apps that track the number of steps. Instead, parental perceived financial security was a significant predictor of using these apps. As mentioned earlier, apps that count the number of steps are mostly built-in apps in the latest smartphones. Regardless of parental involvement, adolescents whose parents perceive higher financial security could be more likely to have, and already use, these apps on their mobile phones. Therefore, the role of parental online health information–seeking mediation might have been attenuated for these types of apps. Nevertheless, overall, our findings highlight parents as significant role models for their children’s adoption of, and engagement with, mHealth apps when they actively mediate their online health information–seeking behaviors.

### Limitations and Future Research

The limitations of this study should be considered when interpreting its findings. The cross-sectional design of the study limits the interpretability of the results; for instance, although physical activity was associated with mHealth app use, we cannot determine whether it is a consequence of using mHealth apps or a precursor for engagement and use patterns. The assessments were based on self-reports. Thus, we could not control the response characteristics of the participants. The data were collected when lockdown regulations regarding the COVID-19 epidemic were in force in the Czech Republic. It is possible that the frequency of using mHealth apps might have been influenced by these regulations. The majority of the parents who completed the questionnaires were mothers. Therefore, the results should not be generalized to parental dyads. On the basis of *t* test statistics, we identified significant differences between users and nonusers of mHealth apps. However, effect sizes for the observed differences were either negligible or in the small range, limiting the generalizability of the findings. Therefore, determining the factors that differentiate the groups with larger effect sizes are required. In addition to group-based differences, we conducted logistic regression analysis to predict adolescents’ mHealth app use, controlling for the roles of sociodemographic, adolescent, and parental factors hierarchically. We also examined the predictors of mHealth app use by type of app. Overall, this study provides a comprehensive overview of how adolescent and parental factors, including sociodemographics, digital skills, and health indicators, were associated with mHealth app use in a representative sample of adolescents in Europe.

Future research could determine how adolescents’ use of mHealth apps might be related to their health outcomes and behaviors longitudinally. In addition, a previous study identified individual factors that may cause harm because of the potentially maladaptive use of mHealth apps in an adult sample [45]. Another study raised concerns about the long-term impact of the use of nutrition and fitness apps on young adults at risk of maladaptive eating and excessive exercise [46]. Therefore, future research could also investigate those factors that are related to potentially harmful psychological and health outcomes of adolescents’ mHealth app use.

### Conclusions

This study examined mHealth app use of adolescents in a representative sample. It showed that older age, higher eHealth literacy skills, and physical activity were related to adolescents’ use of mHealth apps. Girls were more likely to adopt mHealth app technologies, and they tracked their physical activity with apps more regularly than boys. In addition, adolescents who reported higher BMI, health anxiety, and lower sleep quality used health apps more frequently to manage weight, eating behaviors, and health. Finally, we showed initial evidence for the significant role played by parental mediation of online health information–seeking behaviors in adolescents’ adoption and use of mHealth apps. These findings facilitate a more comprehensive understanding of health technology use by adolescents.

## Abbreviations

mHealth: mobile health

## References

[1] Anderson M, Jiang J (2018). Teens, Social Media and Technology 2018.

[2] Smahel D, Machackova H, Mascheroni G, Dedkova L, Staksrud E, Ólafsson K (2020). EU Kids Online 2020: survey results from 19 countries.

[3] Rideout V, Fox S (2018). Digital health practices, social media use, and mental well-being among teens and young adults in the U.S.

[4] Rideout V, Fox S, Peebles A, Rob MB (2021). Coping with COVID-19: how young people use digital media to manage their mental health.

[5] Virella Pérez YI, Medlow S, Ho J, Steinbeck K (2019). Mobile and web-based apps that support self-management and transition in young people with chronic illness: systematic review. J Med Internet Res.

[6] Emberson MA, Lalande A, Wang D, McDonough DJ, Liu W, Gao Z (2021). Effectiveness of smartphone-based physical activity interventions on individuals' health outcomes: a systematic review. Biomed Res Int.

[7] Brannon EE, Cushing CC (2015). A systematic review: is there an app for that? Translational science of pediatric behavior change for physical activity and dietary interventions. J Pediatr Psychol.

[8] Böhm B, Karwiese SD, Böhm H, Oberhoffer R (2019). Effects of mobile health including wearable activity trackers to increase physical activity outcomes among healthy children and adolescents: systematic review. JMIR Mhealth Uhealth.

[9] Kouvari M, Karipidou M, Tsiampalis T, Mamalaki E, Poulimeneas D, Bathrellou E, Panagiotakos D, Yannakoulia M (2022). Digital health interventions for weight management in children and adolescents: systematic review and meta-analysis. J Med Internet Res.

[10] Bond SJ, Parikh N, Majmudar S, Pin S, Wang C, Willis L, Haga SB (2022). A systematic review of the scope of study of mHealth interventions for wellness and related challenges in pediatric and young adult populations. Adolesc Health Med Ther.

[11] Olla P, Shimskey C (2015). mHealth taxonomy: a literature survey of mobile health applications. Health Technol.

[12] Smahel D, Elavsky S, Machackova H (2019). Functions of mHealth applications: a user's perspective. Health Informatics J.

[13] Ng K, Tynjälä J, Kokko S (2017). Ownership and use of commercial physical activity trackers among finnish adolescents: cross-sectional study. JMIR Mhealth Uhealth.

[14] Tran BX, Zhang MW, Le HT, Nguyen HD, Nguyen LH, Nguyen QL, Tran TD, Latkin CA, Ho RC (2018). What drives young vietnamese to use mobile health innovations? Implications for health communication and behavioral interventions. JMIR Mhealth Uhealth.

[15] De Cock N, Vangeel J, Lachat C, Beullens K, Vervoort L, Goossens L, Maes L, Deforche B, De Henauw S, Braet C, Eggermont S, Kolsteren P, Van Camp J, Van Lippevelde W (2017). Use of fitness and nutrition apps: associations with body mass index, snacking, and drinking habits in adolescents. JMIR Mhealth Uhealth.

[16] Liu D, Maimaitijiang R, Gu J, Zhong S, Zhou M, Wu Z, Luo A, Lu C, Hao Y (2019). Using the unified theory of acceptance and use of technology (UTAUT) to investigate the intention to use physical activity apps: cross-sectional survey. JMIR Mhealth Uhealth.

[17] Cho J, Park D, Lee HE (2014). Cognitive factors of using health apps: systematic analysis of relationships among health consciousness, health information orientation, eHealth literacy, and health app use efficacy. J Med Internet Res.

[18] Nutbeam D (2000). Health literacy as a public health goal: a challenge for contemporary health education and communication strategies into the 21st century. Health Prom Int.

[19] Sørensen K, Van den Broucke S, Fullam J, Doyle G, Pelikan J, Slonska Z, Brand H, (HLS-EU) Consortium Health Literacy Project European (2012). Health literacy and public health: a systematic review and integration of definitions and models. BMC Public Health.

[20] Levin-Zamir D, Bertschi I, Okan O, Bauer U, Levin-Zamir D, Pinheiro P, Sørensen K (2019). Media health literacy, eHealth literacy and health behaviour across the lifespan: current progress and future challenges. International Handbook of Health Literacy: Research, Practice and Policy Across the Life-span.

[21] Ernsting C, Dombrowski SU, Oedekoven M, O Sullivan JL, Kanzler M, Kuhlmey A, Gellert P (2017). Using smartphones and health apps to change and manage health behaviors: a population-based survey. J Med Internet Res.

[22] Weiser EB (2001). The functions of internet use and their social and psychological consequences. Cyberpsychol Behav.

[23] Reich SM, Starks A, Santer N, Manago A (2021). Brief report–modeling media use: how parents’ and other adults’ posting behaviors relate to young adolescents’ posting behaviors. Front Hum Dyn.

[24] Vaala SE, Bleakley A (2015). Monitoring, mediating, and modeling: parental influence on adolescent computer and internet use in the United States. J Child Media.

[25] Livingstone S, Ólafsson K, Helsper E, Lupiáñez-Villanueva F, Veltri G, Folkvord F (2017). Maximizing opportunities and minimizing risks for children online: the role of digital skills in emerging strategies of parental mediation. J Commun.

[26] Chang F, Chiu C, Chen P, Miao N, Lee C, Chiang J, Pan Y (2015). Relationship between parental and adolescent eHealth literacy and online health information seeking in Taiwan. Cyberpsychol Behav Soc Netw.

[27] Gulec H, Kvardova N, Smahel D (2022). Adolescents' disease- and fitness-related online health information seeking behaviors: the roles of perceived trust in online health information, eHealth literacy, and parental factors. Comput Human Behav.

[28] Hong IK (2021). Parenting in the digital age: an examination of predictors and outcomes of parental mediation [Doctoral dissertation].

[29] Konok V, Bunford N, Miklósi A (2019). Associations between child mobile use and digital parenting style in Hungarian families. J Child Media.

[30] Mana M (2022). Informační Společnost V Číslech 2022 Česká Republika a Eu.

[31] Norman CD, Skinner HA (2006). eHEALS: the eHealth literacy scale. J Med Internet Res.

[32] Longley SL, Watson D, Noyes R (2005). Assessment of the hypochondriasis domain: the multidimensional inventory of hypochondriacal traits (MIHT). Psychol Assess.

[33] Dedkova L, Smahel D (2019). Online parental mediation: associations of family members’ characteristics to individual engagement in active mediation and monitoring. J Family Issues.

[34] (2021). IBM SPSS Statistics for Windows, Version 28.0.

[35] Drake C, Cannady M, Howley K, Shea C, Snyderman R (2019). An evaluation of mHealth adoption and health self-management in emerging adulthood. AMIA Annu Symp Proc.

[36] Hong H (2011). An extension of the extended parallel process model (EPPM) in television health news: the influence of health consciousness on individual message processing and acceptance. Health Commun.

[37] Hong H, Chung W (2022). Integrating health consciousness, self-efficacy, and habituation into the attitude-intention-behavior relationship for physical activity in college students. Psychol Health Med.

[38] Pu B, Zhang L, Tang Z, Qiu Y (2020). The relationship between health consciousness and home-based exercise in China during the COVID-19 pandemic. Int J Environ Res Public Health.

[39] Jungmann SM, Brand S, Kolb J, Witthöft M (2020). Do Dr. Google and health apps have (comparable) side effects? an experimental study. Clin Psychological Sci.

[40] Vaghefi I, Tulu B (2019). The continued use of mobile health apps: insights from a longitudinal study. JMIR Mhealth Uhealth.

[41] Dion J, Blackburn M, Auclair J, Laberge L, Veillette S, Gaudreault M, Vachon P, Perron M, Touchette (2015). Development and aetiology of body dissatisfaction in adolescent boys and girls. Int J Adolesc Youth.

[42] Fergus TA, Limbers CA, Griggs JO, Kelley LP (2018). Somatic symptom severity among primary care patients who are obese: examining the unique contributions of anxiety sensitivity, discomfort intolerance, and health anxiety. J Behav Med.

[43] Stauss HM (2003). Heart rate variability. Am J Physiol Regul Integr Comp Physiol.

[44] Ferranti R, Marventano S, Castellano S, Giogianni G, Nolfo F, Rametta S, Matalone M, Mistretta A (2016). Sleep quality and duration is related with diet and obesity in young adolescent living in Sicily, Southern Italy. Sleep Sci.

[45] Elavsky S, Smahel D, Machackova H (2017). Who are mobile app users from healthy lifestyle websites? Analysis of patterns of app use and user characteristics. Transl Behav Med.

[46] Honary M, Bell BT, Clinch S, Wild SE, McNaney R (2019). Understanding the role of healthy eating and fitness mobile apps in the formation of maladaptive eating and exercise behaviors in young people. JMIR Mhealth Uhealth.

